# Opioids in the Treatment of Chronic Idiopathic Diarrhea in Humans—A Systematic Review and Treatment Guideline

**DOI:** 10.3390/jcm12072488

**Published:** 2023-03-24

**Authors:** Christoffer S. Graven-Nielsen, Cecilie S. Knoph, Tina Okdahl, Katrine L. Høyer, Klaus Krogh, Per M. Hellström, Asbjørn M. Drewes

**Affiliations:** 1 Mech-Sense, Department of Gastroenterology and Hepatology, Aalborg University Hospital, 9000 Aalborg, Denmark; c.gravennielsen@rn.dk (C.S.G.-N.);; 2 Clinical Institute, Aalborg University Hospital, 9000 Aalborg, Denmark; 3 Department of Hepatology and Gastroenterology, Aarhus University Hospital, 9000 Aalborg, Denmark; 4 Danish Cancer Society Centre for Research on Survivorship and Late Adverse Effects after Cancer in the Pelvic Organs, Aarhus University Hospital, 9000 Aarhus, Denmark; 5 Department of Medical Sciences, Uppsala University, 75236 Uppsala, Sweden

**Keywords:** idiopathic, chronic, diarrhea, opioids, guidelines

## Abstract

In patients with chronic idiopathic diarrhea resistant to standard treatment, opioids are often used as rescue therapy. This systematic review investigated opioid effects on gut function in chronic diarrhea. PubMed and Embase were searched regarding effects of opioid agonists on the gastrointestinal tract in humans with chronic or experimentally induced diarrhea. A total of 1472 relevant articles were identified and, after thorough evaluation, 11 clinical trials were included. Generally, studies reported a reduction in stool frequency and an increase in transit time during treatment with the opioid receptor agonists loperamide, asimadoline, casokefamide, and codeine compared with placebo. Loperamide and diphenoxylate significantly improved stool consistency compared with placebo, whereas asimadoline showed no such effects. Compared with placebo, loperamide treatment caused less abdominal pain and urgency. Asimadoline showed no significant subjective improvements, but fedotozine was superior to placebo in reducing abdominal pain and bloating in selected patients. Only two relevant studies were published within the last 20 years, and standardized endpoint measures are lacking. Most trials included few participants, and further evidence is needed from larger, prospective studies. Likewise, consensus is needed to standardize endpoints for stool frequency, transit time, and consistency to conduct future meta-analyses on opioids in management of chronic idiopathic diarrhea.

## 1. Introduction

Pragmatically, chronic diarrhea is defined as a history of three or more loose or liquid bowel movements per day for at least four weeks [1]. This debilitating condition affects up to 5% of the population [2] and has a severe impact on the quality of life in affected individuals [3,4]. Furthermore, chronic diarrhea may hinder a normal everyday life and has major social consequences. Better treatment options for this patient group are therefore highly warranted.

Common underlying causes of chronic diarrhea include gastrointestinal malignancies, inflammatory bowel disease, pancreatic exocrine insufficiency, bile acid malabsorption, bacterial overgrowth, and late effects after radiation therapy or colon resections [5]. Treatment strategies rely on targeted therapy aiming at alleviating the underlying cause of the disease. In some cases, however, the pathogenesis remains unclear or targeted treatment is not available or fails. In such cases, several antidiarrheal agents are available (e.g., fiber supplementation, bile acid resins, anticholinergic medication), but the approach is generally based on trial and error. In cases of chronic diarrhea refractory to standard antidiarrheal therapy, opioids are often used as a rescue in the empiric treatment strategy [5,6]. Generally, first line of treatment is non-pharmacological with dietary interventions, and opioids are recommended as second line of treatment.

Endogenous opioid receptor ligands such as enkephalins, endorphins, and dynorphins facilitate normal regulation of gastrointestinal function through activation of opioid receptors within the alimentary tract. The effects include inhibition of motility, decreased secretory function, and increased sphincter tone. In humans, the distribution of different opioid receptors and subclasses has not been fully elucidated, but the µ-opioid receptors are thought to be of fundamental importance regarding regulation of gastrointestinal function. [7,8,9]. Exogenous opioids such as morphine and morphine-like compounds are able to activate endogenous opioid receptors within the gastrointestinal tract [10] as evidenced by the fact that constipation is the most common adverse effect of opioid therapy for analgetic purposes [11,12].

Even though opioids are recommended as second-line choice for chronic diarrhea, evidence-based knowledge regarding their effects is sparse, and, consequently, clinical practice varies greatly among medical health providers. Thus, the aim of this systematic review was to investigate the literature regarding gastrointestinal effects of opioid agonist treatments. We included both studies in healthy subjects with experimental diarrhea and in patients with chronic diarrhea of unknown cause in order to provide an overview of the evidence behind this treatment strategy. To this end, we propose an evidence-based treatment guideline.

## 2. Methods

### 2.1. Search Strategy

A systematic literature search was performed in PubMed and Embase using controlled vocabulary (diarrhea; analgesic; opioid; opiate; gastrointestinal tract; intestine) and free text words (diarrhea, diarrhoea, opioid*, opiat*, ‘opium tincture’, gastrointest*, intestin*, digestive, colon*, gastri*, bowel). The literature search was performed on 11 March 2021 and again on 13 December 2022. Chain searching was applied to expand the number of included articles.

Title, abstract, and subsequent full text screening was performed by two independent reviewers (CSGH and KLH) using an online tool for blinded inclusion and exclusion of studies (Rayyan Systems) [13]. In case of doubt, a third reviewer (AMD) was consulted.

Chronic diarrhea was defined by the authors as ≥4 weeks of ≥3 stools of a loose consistency per day or by having the diagnosis IBS-D [14].

### 2.2. Inclusion Criteria

The following inclusion criteria were applied: original clinical trials conducted on patients with chronic diarrhea or healthy volunteers with experimentally induced diarrhea; treatment with opioid agonists; one or more of the following outcome measures: bowel movement frequency, stool consistency, transit time, or symptom burden. Only articles in English, Danish, Norwegian, or Swedish were included.

### 2.3. Exclusion Criteria

Studies applying opioid receptor antagonists, mixed agonist–antagonist opioids (e.g., eluxadoline) or endogenous opioids (e.g., enkephalins) as intervention were excluded, because this review focused on exogenous opioids agonist binding to the μ-opioid receptor. Furthermore, mechanistic studies of opioids in, for example, healthy subjects or patients with pain and opioid-induced constipation were not included. Case reports and abstract-only studies were also excluded.

### 2.4. Data Extraction

Data were extracted from eligible studies by two independent reviewers (CSGN and KLH) regarding: study characteristics, participant characteristics, setting, intervention, and outcome measures.

### 2.5. Quality Assessment

Quality assessment was performed by two reviewers (KLH and CSGH) using RoB 2 (risk of bias in randomized trials) and ROBINS-I (risk of bias in non-randomized studies of interventions) [15]. The quality assessment scores were registered using REDCap electronic data capture tools hosted at Aalborg University Hospital, allowing for blinded risk assessment of each article. Unblinding was done after all articles were risk assessed. In cases of disagreement, the highest risk of bias was retained. Studies with moderate or above overall risk of bias were excluded.

## 3. Results

**Figure 1:**
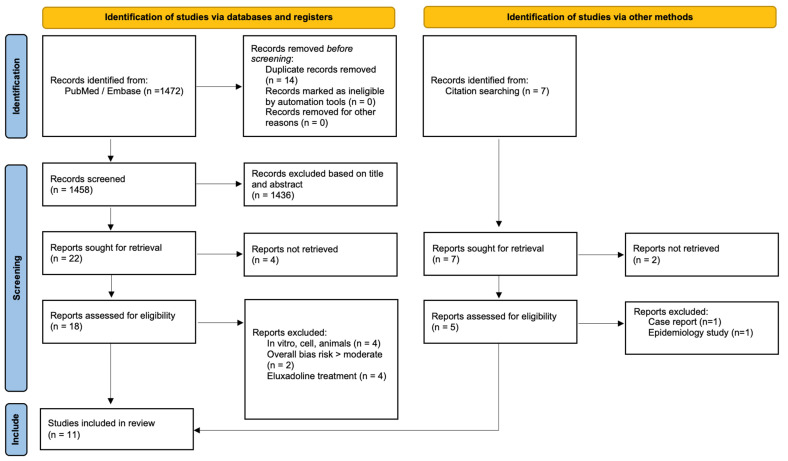
PRISMA flow diagram.

A total of 1472 relevant articles were identified through the systematic literature search and 7 articles were found during subsequent chain searching. After screening of title and abstract, 23 articles were left for full text screening, 11 of which were included in this review (Figure 1).

In 7 of the 11 included articles, the effects of loperamide treatment were investigated with median daily doses of 2–24 mg. Most articles reported an individual optimum of 2–6 mg daily. The articles that investigated the effects of codeine phosphate reported a median daily dose of 30–60 mg. Asimadoline doses reported were median daily doses of 0.15–1.0 mg. The one article that investigated the effect of casokefamide reported a median daily dose of 5.5–16.0 mg.

### 3.1. Opioid Receptor Agonist Effects on Bowel Movement Frequency

A total of nine studies included self-reported stool frequency when an opioid receptor agonist was used (Table 1). Eight of the studies showed that the opioid agonists loperamide, asimadoline, or codeine reduced the stool frequency in a setting of chronic or experimentally induced diarrhea.

Loperamide was reported to give a 36% reduction in stool frequency compared with placebo throughout five weeks of treatment (*p* < 0.001) [19]. A reduction in stool frequency in diarrhea due to ileo-colic disease or resection by loperamide compared with the placebo group was supported by Mainguet et al. [17]. Two other studies showed that loperamide reduced the mean stool frequency per week compared with placebo (loperamide: 11–13 per week vs. placebo: 17–19 per week) [16,18]. Loperamide resulted in a reduction of the daily stool frequency from 1.9 ± 0.2 at baseline to 1.3 ± 0.1 times per day, while placebo remained at 1.9 ± 0.2 times per day [21] In a dose–response study, 2 mg loperamide was superior to the combination of 5 mg of diphenoxylate plus 0.05 mg of atropine sulfate with regards to decreased stool frequency in patients with chronic diarrhea [22]. In line with this, one study on diarrhea-predominant irritable bowel syndrome (IBS-D) found a significant effect on stool frequency of loperamide capsules compared with placebo [20].

A daily dose of 0.5 mg asimadoline reduced stool frequency compared with placebo in IBS-D (asimadoline: 2.3 fewer bowel emptyings per day; placebo: 0.3 fewer emptyings per day) [23]. Similarly, codeine was investigated in lactulose-induced diarrhea in healthy subjects, and resulted in a reduction in daily stool frequency from 2.4 ± 1.0 times per day to 1.2 ± 0.4 times per day [24].

### 3.2. Opioid Receptor Agonist Effects on Transit Time

Five of the 11 included articles investigated the effect of opioids on gastrointestinal transit time (Table 2). The investigational approach varied between studies, with three studies using radiolabeled markers and scintigraphy for assessment of segmental transit times. Two studies measured time-to-rise of exhaled hydrogen concentration after ingestion of lactulose as a measure of oro–cecal transit time, and one study evaluated whole gut transit as the time from ingestion of carmine red to the first appearance of red stools.

Loperamide increased whole gut transit time in patients with chronic diarrhea due to ileo-colic disease or resection compared with placebo (median absolute transit time 2.2 vs. 4.6 h; 40% reduction of stool weight) [17]. In a cohort of patients with diarrhea and IBS, segmental analysis revealed reduced gastric emptying time, while small bowel transit was prolonged, resulting in an increased whole gut transit time during loperamide treatment (56 ± 5 vs. placebo 42 ± 4 h) [21]. A similar pattern of increased gastric emptying and prolonged small bowel and whole gut transit was found in another study investigating the effect of a loperamide precursor (loperamide-N-oxide) on radiation-induced chronic diarrhea [16]. In a lactulose-induced experimental model of diarrhea, casokefamide, a peripherally acting synthetic opioid pentapeptide drug that binds to both μ- and δ-opioid receptors, significantly prolonged oro–cecal transit time in 6 out of 10 healthy participants. The remaining four subjects, however, appeared to be non-responsive to the treatment [25]. In a similar experimental model of diarrhea, codeine significantly delayed mouth-to-ileum transit time of capsule markers from 2.8 ± 1.0 to 5.3 ± 3.2 h, and a further delay of ascending colonic transit time from 5.3 ± 2.5 to 7.4 ± 2.5 h. However, the effect in the descending colon was limited [24].

### 3.3. Opioid Receptor Agonist Effects on Stool Consistency

Seven articles investigated stool consistency in patients with chronic diarrhea following opioid treatment (Table 3). In all studies, consistency was self-reported during treatment. The different studies used varying approaches to evaluate levels of consistency. Thus, two studies [18,21] evaluated consistency as formed or unformed, whereas three other studies [17,20,22] evaluated consistency on a five-level scale (watery—liquid—loose—intermediate—shaped—formed). Two studies evaluated stool consistency on the numeric Bristol Stool Scale of 1–7 [23,26] or 0–100 [19], with the low scores representing hard stools and high scores representing loose or watery stools. 

Generally, treatment with loperamide improved stool consistency compared with placebo. In patients with persistent diarrhea of various etiologies (including inflammatory bowel disease), a decrease in the percentage of unformed stools per week during treatment with loperamide was demonstrated compared with placebo (29 vs. 62 %) [18]. Similarly, improved stool consistency was seen in studies investigating IBS in both cross-over and parallel randomized controlled trials [19,20,21]. Another study [17] found a significant decrease in the median daily number of unformed stools during treatment with loperamide compared with placebo in patients with ileo-colic disease or resection due to Crohn’s disease (1.5 vs. 4.5 daily).

Loperamide was also compared with diphenoxylate in patients with chronic diarrhea of various etiologies, who had all their antidiarrheal medication withdrawn to induce symptoms [22]. This study found an improved stool consistency during treatment with both loperamide and diphenoxylate compared with a drug-free interval. In this context, treatment with loperamide was also reported to be significantly better than diphenoxylate.

One study examined the effects of asimadoline in patients with IBS and found no difference in stool consistency compared with placebo [27]. 

### 3.4. Opioid Receptor Agonist Effect on Subjective Improvement

In total, 6 articles evaluated change in subjective symptoms in patients with chronic diarrhea and treatment with an opioid receptor agonist (Table 4). All articles except one evaluated the response to treatment or placebo by subjective symptom rating—most commonly abdominal pain—using different numerical rating scales [16,19,21,27,28]. Four studies used a rating scale of 3, 4 or 5 points [16,21,27,28], whereas one study used a visual analogue scale of 0–100 [19]. In all rating scales, higher numbers indicated an increased severity of symptoms. The last study evaluated the subjective response to treatment on several symptoms (including abdominal pain) merely as better (+1), unchanged (0) or worse (−1).

Two studies found a reduction of up to 30% in abdominal pain during treatment with loperamide compared with placebo in patients with IBS [19,20]. Conversely, one of these studies observed an increase of nighttime pain in the loperamide group [19], whereas another study found that loperamide was non-superior to placebo [21]. An improvement in urgency symptoms was also found in two studies [20,21]. The subjectively better response to loperamide treatment compared with placebo was similar in patients with chronic radiation enteritis [16]. In patients with IBS-D, a significant improvement in pain relief, pain scores, and pain-free days was seen during treatment with asimadoline compared with placebo [27]. Lastly, one study examined the effects of the opioid κ-receptor agonist fedotozine at different doses (3.5 mg, 15 mg, or 30 mg, all TID) compared with placebo in patients with symptoms characteristic of IBS. In these patients, a superior relief of maximum daily abdominal pain, mean daily pain, and abdominal bloating was found with the highest dose of fedotozine compared with placebo [28].

### 3.5. Adverse Effects

In total, 4 [18,20,22,23] of the 11 included articles reported one to eleven subjects per article having occasional constipation. Four articles [17,18,22,23] reported one to two subjects per article having abdominal pain. Two articles [18,23] reported one to four subjects per article having diarrhea.

## 4. Discussion

In this systematic review we examined the evidence of opioid receptor agonists in treatment of idiopathic chronic or experimental diarrhea. Overall, evidence was sparse and outdated with a lack of standardized methods for outcome reporting. Nonetheless, studies generally reported a reduction of stool frequency and an increase in transit time during treatment with loperamide. Asimadoline produced a decrease in stool frequency compared with placebo, but only in patients with diarrhea-predominant IBS. In addition, casokefamide and codeine were found to delay transit time in chronic or experimentally induced diarrhea. This is in keeping with previous findings that opioids have an inhibitory effect on gastrointestinal motility [29]. Loperamide also significantly improved stool consistency in patients with chronic diarrhea and was found to be superior to diphenoxylate. On the other hand, asimadoline showed no significant effect on stool consistency in patients with IBS-D. This underlines the potential of opioid receptor agonists on secretion and absorption of water and electrolytes in the gut. Abdominal pain and urgency were improved during treatment with loperamide, but one study also found an increase in nighttime pain. Asimadoline produced no significant subjective improvement, but fedotozine was superior to placebo in reducing abdominal pain and bloating in patients with IBS.

### 4.1. Opioid Agonistic Effects on the Gastrointestinal Tract

As expected, we found that treatment with opioid receptor agonists increased the fecal consistency, increased the gastrointestinal transit time, and decreased bowel movement frequency in patients with chronic or experimentally induced diarrhea. Physiologically, the intestinal fluid balance is essential in the establishment of an intestinal environment ideal for processing of nutrients. In the gut, the submucosal myenteric plexus controls the local secretory and absorptive activity [9,30]. Hence, serotonin, acetylcholine, and vasoactive intestinal peptide are released from the neurons and activate intracellular mechanisms in mucosa cells, which again activate epithelial cellular chloride channels in the epithelium. As chloride ions move from the enterocyte cytoplasm into the gut lumen, water follows via the osmotic gradient [31,32,33].

The pathophysiology of diarrhea often depends on a dysregulation of the enteral water and electrolyte equilibrium, which results in increased fecal volume with increased water content [34]. Opioids bind to the secretomotor neurons in the submucosal plexus and suppress neurotransmitter release, resulting in decreased chloride and water secretion [32]. In this way, secretion and absorption of water in the gut are affected by opioids [7,35,36]. In diarrhea, increased fecal volume causes mechanoreceptor activation (intrinsic reflexes) with increasing propulsive gut movements [7,34]. A decrease in fecal volume has a negative impact on motility by reducing activity of intrinsic reflex arcs [7]. Furthermore, opioid receptor activation results directly in an increase of smooth muscle tone with decreased propulsive activity, which enhances water uptake from the gut. The opioid receptor activation is mainly exerted via μ-receptors in the myenteric plexus [37]. The opioid receptors are believed to activate an outward potassium conductance, involving adenylate cyclase and decreased opening of calcium channels, with a reduction in cell excitability and neurotransmission [38]. Hence, opioid administration leads to an increase of gastric tone [39,40,41,42], with slowing of transit times through the stomach, small bowel, and colon [43,44,45]. In line with these findings, we previously showed that the opioid agonists oxycodone and tapentadol increased colonic as well as whole gut transit time, resulting in a reduction of water secretion in healthy controls [46,47,48,49,50,51,52]. Opioids also increase anal sphincter tone, potentially alleviating symptoms of fecal incontinence [18,53].

Drugs such as opium tincture are also used in patients with diarrhea resistant to standard treatment. So far, no studies have investigated medicinal grade opium tincture in clinical trials, and support for its effect is based on expert consensus. A recent, yet unpublished, study conducted by our research group found an increase in colonic and whole gut transit time with reduction of daily stools in healthy controls treated with medicinal grade opium tincture. This underlines the importance of new evidence from larger, prospective studies within this field.

### 4.2. Safety and Potential for Abuse

Although antidiarrheal agents such as loperamide are generally considered safe, there are potentially jeopardizing effects on cardiac function, especially in high doses and when taken in combination with drugs metabolized by the CYP3A4 isoenzyme [54]. Concurrent administration with CYP3A4-inhibiting drugs may elevate loperamide concentrations. Hence, ventricular arrhythmia, prolonged QST-complexes, and disrupted QTc-intervals have been shown in patients with loperamide abuse (defined as supratherapeutic dosing, without a prescription) [55]. When initiating treatment with opioids, potential abuse is always a concern. Loperamide has an insignificant central nervous system penetration at therapeutic oral doses and is generally considered safe to prescribe for patients with chronic idiopathic diarrhea. Contrary to this, supratherapeutic oral doses of loperamide may affect the central nervous system—providing opportunity for abuse [56]. One review even identified loperamide misuse as contributing to several deaths [57]. Codeine also carries a potential for abuse and has likewise been identified as contributing to drug-related deaths in some cases [58]. For other opioids used to treat chronic diarrhea, the potential for abuse is vastly unknown.

### 4.3. Guidelines for Treatment of Chronic Idiopathic Diarrhea

**Figure 2:**
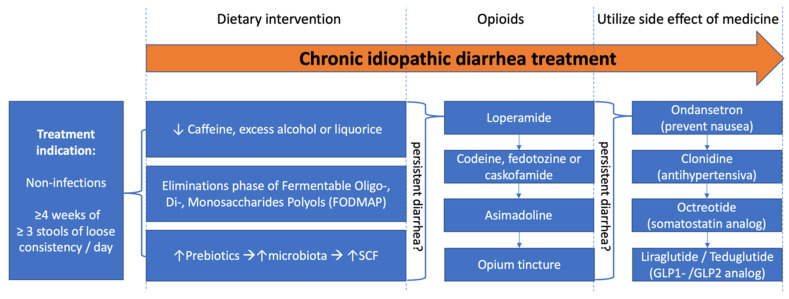
Clinical treatment algorithm used to select treatment level for patients with chronic idiopathic diarrhea.

This review showed that standardized guidelines are missing, and we have a knowledge gap as regards treatment of chronic idiopathic diarrhea. Several antidiarrheal agents are available for this, but no specific treatment algorithm has yet been implemented (Figure 2). Generally, first line of treatment is non-pharmacological with dietary interventions, such as reducing consumption of lactose, artificial sweeteners, alcohol sugars, caffeine, licorice, and excess alcohol. Consultancy by a dietician can be helpful and assist the patient in avoiding non-absorbable carbohydrates that rapid pass the small intestine and challenge the colon with an osmotic load, as well as extensive fermentation with production of gas in the colon. Prebiotics are degraded by the gut microbiota metabolism to produce short-chain fatty acids lining the gut lumen and are absorbed into the bloodstream. A few of these prebiotics have been shown to prevent and be of value in treatment of some types of diarrhea [59,60]. Bulking agents such as psyllium can also be of benefit in many cases. In cases of chronic diarrhea resistant to dietary interventions, opioids may be applied as a rescue in an empiric treatment strategy. Based on the summary of findings in this review, the evidence for treatment with loperamide is the most substantiated. Codeine, fedotozine and casokefamide also improved chronic diarrhea although evidence is sparse. Asimadoline showed potential in improving chronic diarrhea, but only in a subgroup of patients. It should be emphasized that fedotozine and asimadoline are still considered experimental medications, although they can be bought online.

As a second-line therapeutic step, expert consensus suggests that patients may also benefit from opium tincture, which has rapid onset and is not dependent on the release of the active drug from a tablet or capsule. The third-line step in the empiric treatment strategy are medicines with specific off-target antidiarrheal effects. One such drug is ondansetron, which has a clear antisecretory component that can be used, often with constipating effects, in oncology treatment. Clonidine exerts increased negative feedback on the release of neurotransmitters through agonistic action on presynaptic α_2_-adrenergic autoreceptors. Octreotide dampens motor and secretory activity in the gut, often experienced as constipation in the treatment of acromegaly and HIV-diarrhea. The more recent glucagon-like peptide-1 receptor agonists slow gastrointestinal motility and secretion, and glucagon-like peptide-2 receptor agonists promote water absorption because of mucosal growth with increased capacity of water and electrolyte absorption.

### 4.4. Strengths and Limitation

Several measures were taken to minimize bias according to the PRISMA guidelines. However, this study has some limitations. Throughout the analysis of the included articles, the outcome parameters showed a great degree of heterogenicity, which underlines the lack of standardized reporting within this field. Furthermore, the articles included for this review range from publication years 1977 to 2008, and only two articles were published within the last 20 years. This emphasizes the need for new up-to-date investigations within this field.

## 5. Conclusions

Opioid receptor agonists have beneficial effects in treatment of chronic idiopathic diarrhea, but evidence is based on data from a limited number of clinical trials. Most reports are old and included relatively few patients or healthy controls. In fact, most of our knowledge is empirical and based on ancient expert opinions, which can question the basis for treatment recommendations. Before new randomized clinical studies are designed, a consensus is needed to standardize endpoints for stool frequency, transit time, and consistency in order to conduct future meta-analyses of the efficacy of opioids in management of idiopathic chronic diarrhea.

## Figures and Tables

**Table 1:** Stool frequency studies.

Author, Year	Study Design	Study Population	Inclusion Criteria	Investigational Product vs. Comparator	Main Findings
E. K. Yeoh, 1993 [16]	Randomized, double-blind, cross-over study	Patients with chronic radiation enteritis (*n* = 20) + Healthy subjects (*n* = 18)	>14 stools/week	Loperamide oxide vs. placebo	Loperamide reduced 31% to a mean of 13/week compared with placebo 19/week (*p* < 0.001)
P. Mainguet, 1977 [17]	Randomized, double-blind, cross-over study	Patients with ileo-colic disease or resection (*n* = 18)	>3 months of diarrhea	Loperamide vs. placebo	1.5 liquid + loose stool/day (loperamide) 4.5 liquid + loose stool/day (placebo) (*p* < 0.001)
M. Read, 1982 [18]	Randomized, double-blind, cross-over study	Patients with chronic diarrhea and fecal incontinence (*n* = 26)	>3 months of diarrhea and complained of episodes of fecal incontinence and severe urgency sufficient to limit lifestyle	Loperamide vs. placebo	Loperamide reduced 35% to a mean of 11/week compared with placebo 17/week (*p* < 0.001)
P.S. Efskind, 1996 [19]	Randomized, double-blind, parallel study	Patients with symptoms of IBS (*n* = 69) + Healthy subjects (*n* = 33)	Weekly symptoms >3 months of abdominal pain and changing stool pattern and consistency	Loperamide vs. placebo	36% reduction in stool frequency compared with placebo and with baseline throughout 5 weeks of treatment (*p* < 0.001)
B. Lävo, 1987 [20]	Randomized, double-blind, parallel study	Patients with symptoms of IBS (*n* = 21)	>3 months of diarrhea with no demonstrable organic bowel disease	Loperamide vs. placebo	Loperamide promising with respect to decreased stool frequency, no difference between treatments
P.A. Cann, 1984 [21]	Randomized, double-blind, cross-over study	Patients with symptoms of IBS (*n* = 28)	Symptoms (abdominal pain and bowel disturbances) present >6 months and >3 days/week	Loperamide vs. placebo	1.3 ± 0.1 stool/day (loperamide) 1.9 ± 0.2 stool/day (placebo) (*p* < 0.001)
W. Pelemans, 1993 [22]	Randomized, double-blind, cross-over study	Patients with colectomy and ileostomy and documented chronic diarrhea of diverse origin	>3 unformed stools/day for 3 consecutive days	Loperamide and diphenoxylate vs. drug free	1.4 liquid + loose stool/day (loperamide) 2.7 liquid + loose stool/day (diphenoxylate) 4.6 liquid + loose stool/day (drug-free mean) (*p* < 0.001)
A.W. Mangel, 2008 [23]	Randomized, double-blind, parallel study	Patients with IBS-D diagnosis (*n*~198)	Meeting Rome II Criteria for IBS and have predominant symptoms of diarrhea	Asimadoline vs. placebo	2.3 stool/day (asimadoline) 2.6 stool/day (placebo) (*p* < 0.005)
L. Barrow, 1993 [24]	Randomized, cross-over study	Healthy subjects (*n* = 12)	Lactulose-induced diarrhea	Codeine phosphate	1.2 stool/day (lactulose + codeine) 2.4 stool/day (lactulose only) (*p* < 0.01)

**Table 2:** Stool consistency studies.

Author, Year	Study Design	Study Population	Inclusion Criteria	Investigational Product vs. Comparator	Main Findings
P. Mainguet, 1977 [17]	Randomized, double-blind, cross-over study	Patients with ileo-colic disease or resection (*n* = 18)	>3 months of diarrhea	Loperamide vs. placebo	1.5 unformed stools/day (loperamide) 4.5 unformed stools/day (placebo) (*p* < 0.001)
M. Read, 1982 [18]	Randomized, double-blind, cross-over study	Patients with chronic diarrhea and fecal incontinence (*n* = 26)	>3 months diarrhea with episodes of fecal incontinence and severe urgency limit lifestyle	Loperamide vs. placebo	29% unformed stools/week (loperamide) 62% unformed stools/week (placebo) (*p* < 0.001)
P.S. Efskind, 1996 [19]	Randomized, double-blind, parallel-group study	Patients with symptoms of IBS (*n* = 69) + Healthy subjects (*n* = 33)	Weekly symptoms >3 months abdominal pain and changing stool pattern and consistency	Loperamide vs. placebo	Loperamide improved stool consistency compared with placebo
B. Lävo, 1987 [20]	Randomized, double-blind study	Patients with symptoms of IBS (*n* = 21)	>3 months of diarrhea with no demonstrable organic bowel disease	Loperamide vs. placebo	Loperamide reduced weekly number of unformed stools compared with placebo
P.A. Cann, 1984 [21]	Randomized, double-blind, cross-over study	Patients with symptoms of IBS (*n* = 28)	Symptoms (abdominal pain and bowel disturbances) present >6 months and >3 days/week	Loperamide vs. placebo	Loperamide reduced weekly percentage of unformed stools compared with placebo
W. Pelemans, 1993 [22]	Randomized, double-blind, cross-over study	Patients with colectomy and ileostomy and chronic diarrhea of diverse origin	>3 unfirmed stools/day for 3 consecutive days	Loperamide and diphenoxylate	Loperamide and diphenoxylate improved stool consistency; loperamide significantly better than diphenoxylate
A.W. Mangel, 2008 [23]	Randomized, double-blind study	Patients with IBS-D diagnosis (*n*~198)	Rome II criteria of IBS and predominant symptoms of diarrhea	Asimadoline vs. placebo	No improvement of fecal consistency in patients on asimadoline at any dose vs. placebo

**Table 3:** Transit time studies.

Author, Year	Study Design	Study Population	Inclusion Criteria	Investigational Product vs. Comparator	Main Findings
P. Mainguet, 1977 [17]	Randomized, double-blind, cross-over study	Patients with ileo-colic disease or resection (*n* = 18)	>3 months of diarrhea	Loperamide vs. placebo	4.6 h whole gut transit (loperamide) 2.2 h whole gut transit (placebo) (*p* < 0.001)
E.K. Yeoh, 1993 [16]	Randomized, double-blinded, cross-over study	Patients with chronic radiation enteritis (*n* = 20) + Healthy subjects (*n* = 18)	>14 stools/week	Loperamide oxide vs. placebo	Loperamide oxide decreased gastric emptying time, and increased small bowel and whole gut transit time compared with placebo Gastric emptying time decreased with loperamide in healthy subjects
P.A. Cann, 1984 [21]	Randomized, double-blind, cross-over study	Patients with symptoms of IBS (*n* = 28)	Symptoms (abdominal pain and bowel disturbances) present >6 months and >3 days/week	Loperamide vs. placebo	Loperamide decreased gastric emptying time, and increased small bowel and whole gut transit time compared with placebo 56 ± 5 h whole gut transit (loperamide) 42 ± 4 h whole gut transit (placebo) (*p* < 0.01)
E. Schulte-Frohlinde, 2000 [25]	Prospective, open dose–response study	Healthy male volunteers (*n* = 10)	No diarrhea	Casokefamide vs. placebo	Casokefamide showed a trend toward prolongation of oro–cecal transit time
L. Barrow, 1993 [24]	Randomized, cross-over study	Healthy subjects (*n* = 12)	Lactulose-induced diarrhea	Codeine phosphate	Codeine increased mouth-to-ileal and colonic transit time primarily in the ascending colon 5.3 ± 3.2 h whole gut transit (codeine) 2.8 ± 1.0 h whole gut transit (placebo) (*p* < 0.02)

**Table 4:** Subjective impression of improvement studies.

Author, Year	Study Design	Study Population	Inclusion Criteria	Investigational Product vs. Comparator	Main Findings
E.K. Yeoh, 1993 [16]	Randomized, double-blinded, cross-over study	Patients with chronic radiation enteritis (*n* = 20) + Healthy subjects (*n* = 18)	>14 stools/week	Loperamide oxide vs. placebo	Gastrointestinal symptoms not different between loperamide and placebo
P.A. Cann, 1984 [21]	Randomized, double-blind, cross-over study	Patients with symptoms of IBS (*n* = 28)	Symptoms (abdominal pain and bowel disturbances) >6 months and >3 days/week	Loperamide vs. placebo	Diarrhea, urgency and borborygmi significantly improved during loperamide compared with placebo
P.S. Efskind, 1996 [19]	Randomized, double-blind, parallel-group study	Patients with symptoms of IBS (*n* = 69) + Healthy subjects (*n* = 33)	Weekly symptoms >3 months of abdominal pain and changing stool pattern and consistency	Loperamide vs. placebo	Loperamide reduced pain intensity, but increased nighttime pain compared with placebo
B. Lävo, 1987 [20]	Randomized, double-blind study	Patients with symptoms of IBS (*n* = 21)	>3 months of diarrhea with no demonstrable organic bowel disease	Loperamide vs. placebo	Less urgency and pain with loperamide compared with placebo
A.W. Mangel, 2008 [23]	Randomized, double-blind study	Patients with IBS-D diagnosis (*n*~198)	Rome II criteria for IBS and predominant symptoms of diarrhea	Asimadoline vs. placebo	Asimadoline relieved IBS pain and diarrhea compared with placebo
M. Dapoigny (9) [28]	Randomized, double-blind, parallel-group study	Patients with symptoms suggestive of IBS diagnosis (*n* = 313)	Abdominal pain ≥moderate intensity and ≥2 additional gastrointestinal symptoms	Fedotozine vs. placebo	Adominal pain and bloating significantly reduced with fedotozine compared with placebo

## Data Availability

No new data were created or analyzed in this study. Data sharing is not applicable in this article.
